# A real-world comparison of apnea–hypopnea indices of positive airway pressure device and polysomnography

**DOI:** 10.1371/journal.pone.0174458

**Published:** 2017-04-05

**Authors:** Ritwick Agrawal, Julie A. Wang, Anita G. Ko, Joanne E. Getsy

**Affiliations:** 1 Pulmonary, Critical Care and Sleep Medicine Section, Baylor College of Medicine, Houston, TX, United States of America; 2 Division of Pulmonary, Critical Care and Sleep Medicine, Drexel University College of Medicine, Philadelphia, PA, United States of America; University of Rome Tor Vergata, ITALY

## Abstract

The apnea hypopnea index (AHI) reported by positive airway pressure (PAP) device is widely used in clinical practice, yet its correlation with standardized AHI obtained during the sleep study is not established. The current study was conducted to investigate the correlation between AHI estimated by the PAP device and reported on the smart card with the AHI found during the PAP polysomnography (PSG) in the “real world” setting at an academic sleep center. We retrospectively reviewed the medical records of 280 patients who underwent a PAP titration PSG at Drexel sleep center, and were later prescribed a PAP device. The AHI was categorized in clinically relevant subgroups (as AHI ≤5 and AHI >5). The AHI at the final pressure on the PSG and the average AHI from the prescribed PAP device were compared. The results showed that in the majority (77.3%) of patients (126 of 163), the AHI from both PAP device and PSG correlated well and were in the same category (AHI ≤5 and AHI >5 respectively). The majority of patients (80.7%) with PSG AHI of <5 had PAP device AHI <5 as well. By contrast, if PSG AHI was >5, 61.5% patients reported good control, with AHI <5 on PAP device AHI. We conclude that in a majority of patients who were optimally titrated in the sleep laboratory, the PAP device continued to show optimal control at home.

## Introduction

Positive airway pressure (PAP) devices are the cornerstone of obstructive sleep apnea syndrome (OSAS) management. In addition to providing therapy for OSAS, they also assess the PAP efficacy and adherence. These devices monitor the hours of usage of device, mask leakage and efficacy of PAP therapy[1]. The efficacy of PAP therapy is usually reported as “residual” apnea hypopnea index (AHI). While the nomenclature of apnea hypopnea index is same for both PAP device AHI and the polysomnography (PSG) AHI, the definitions vary significantly. The AHI determined during a PSG is based on standardized definitions recommended by the American Academy of Sleep Medicine, while the AHI reported by the PAP device, usually with the “smart card” from the machine, is based on a non-standard proprietary definition of the device manufacturer[2]. A consensus statement from American Thoracic Society noted that the AHI data from the PAP device tracking systems are not easy to interpret due to differences in the definition of AHI across various manufacturers[1], and the need for further studies was noted. Furthermore, it has been shown that when PAP AHI from automatic and manual scoring was correlated; it was high for apneic events while the correlation for hypopnea was poor[3].

While the definitions of AHI reported by various devices and the PSG differ, in day-to-day practice clinicians have to utilize the AHI obtained by these different definitions to make management decisions. The current study was therefore, conducted to investigate the correlation between AHI estimated by the PAP device and reported on the smart card with the AHI found during the PAP PSG in the “real world” setting at an academic sleep center. The importance of real-world research in respiratory medicine is increasingly recognized and such studies are recommended in conjunction with classical randomized control trials to provide a fuller picture of outcomes[4].

## Methods

### Subjects

This was a retrospective review of consecutive patients seen between August 2012 and June 2013 who underwent a Constant Positive Airway Pressure (CPAP) titration, a Bilevel Positive Airway Pressure (BPAP) titration, or a split night study at a university-based sleep center in Philadelphia, PA. The study was approved by Drexel university institutional review board (protocol number 1306002128) and a waiver of consent was granted. All patients included in the study were evaluated before the sleep study at the Drexel Sleep Center. Demographic and anthropometric data, vital signs, and body mass index (BMI) were recorded during the initial visit. In patients with a suspicion of obstructive sleep apnea, a diagnostic PSG was performed. Furthermore, if the study was positive for OSAS as defined by the AASM guidelines[5], a technologist-attended PAP titration was performed. Among the patients older than 18 years of age who underwent a PAP titration during the study period, 280 were considered for inclusion in the study. Of those, 117 patients were excluded for the following reasons: failure to follow up within 3 months, weight gain or weight loss of more than 10% on the follow-up visit, recommendation of adaptive servoventilation or autotitrating PAP therapy after the PAP titration, inability of the PAP equipment to report the AHI, PAP pressure adjustment since the sleep study, or major leak for more than 20 minutes on average each night as noted on the smart card.

### Sleep studies

The PSG was performed using standard PSG montage, using SomnoStar software (CareFusion Corp., San Diego, CA). AASM criteria were used for defining apnea and hypopnea. An obstructive apnea is defined as a decrease in the airflow by ≥90% for at least 10 seconds. For hypopnea, either the recommended or the additional definition for AASM-recommended or alternative scoring was used, depending on the patient’s insurance specification[2]. The studies were scored by an experienced registered PSG technologist and interpreted by a board-certified sleep specialist. The PAP titration was performed using the Respironics OmniLab titration system (Philips Healthcare, Andover, MA) utilizing the standard PAP titration montage. The data were collected and analyzed using SomnoStar software.

### Follow-up

After the PAP titration, the patients received PAP equipment. The patients were scheduled to follow up within 90 days of PAP therapy to review adherence, tolerance, and effectiveness of treatment. At the follow-up visit, the patient’s objective PAP adherence and efficacy data were downloaded from the smart card and reviewed.

### Statistical methods

Study data were collected and managed using Research Electronic Data Capture (REDCap) tools hosted at Drexel University. The baseline demographics and outcome measures between groups were compared using two-tailed unpaired t-tests for normally distributed data and Mann-Whitney tests for skewed or non-normally distributed data as appropriate. Correlations between numeric variables were assessed using the Spearman coefficient. When an AHI cutoff of ≤5 was used for sensitivity and specificity calculation, the continuity-corrected χ^2^ test was used. In subgroup analysis, an uncorrected χ^2^ test was used for comparing statistical significance in nominal variables, and linear-trend χ^2^ was used for identifying statistically significant trends. All data analysis was performed using SPSS software version 20 (IBM Corp., Armonk, NY).

## Results

### Patient population

Baseline characteristics and demographic data for the 163 patients are listed in Table 1. Participants were primarily middle aged, obese women and had moderate to severe obstructive sleep apnea. Descriptive statistics for the PSG and PAP device data are included in Table 2.

**Table 1:** Characteristics of study population (n = 163).

Variable	Mean ± SD
**Age, y**	50.5 ± 12.8
**BMI, kg/m**^**2**^	38 ± 9.3
**Sex**	** **
Male	68 (42%)
Female	95 (58%)
**Race**	** **
White	58 (36%)
African American	83 (51%)
Hispanic	11 (7%)
Asian	6 (4%)
Other	5 (3%)
**Severity of OSAS**	** **
Mild	37 (23%)
Moderate	51 (31%)
Severe	75 (46%)
**Type of device**	
CPAP	130 (80%)
BPAP	33 (20%)
**Device manufacturer**	
Respironics ^a^	150 (92%)
Resmed ^b^	13 (8%)

BMI, body mass index.OSAS, obstructive sleep apnea. CPAP, continuous positive airway pressure, BPAP, bilevel positive airway pressure

^a^ Philips Healthcare, Andover, MA

^b^ Resmed Corp., San Diego, CA

**Table 2:** Descriptive statistics for PSG and PAP device data (n = 163).

Variable	Mean ± SD
**PAP titration variables**	
Optimal IPAP (cm of water)	12.2 ± 4.7
AHI	1.5 ± 2
Lowest saturation at optimal PAP (%)	92.8 ± 2.1
Time of recording (min) at the effective pressure	78 ± 58
**PAP device variables**	
Optimal IPAP (cm of water)	12.2 ± 4.7
AHI	3.1 ± 2.7
Days of reported data	32.4 ± 20
Median daily usage (min)	283 ± 142

IPAP, inspiratory positive airway pressure; PAP, positive airway pressure; PSG, polysomnography; AHI, apnea–hypopnea index.

### Correlation and classification of groups

**Fig 1:**
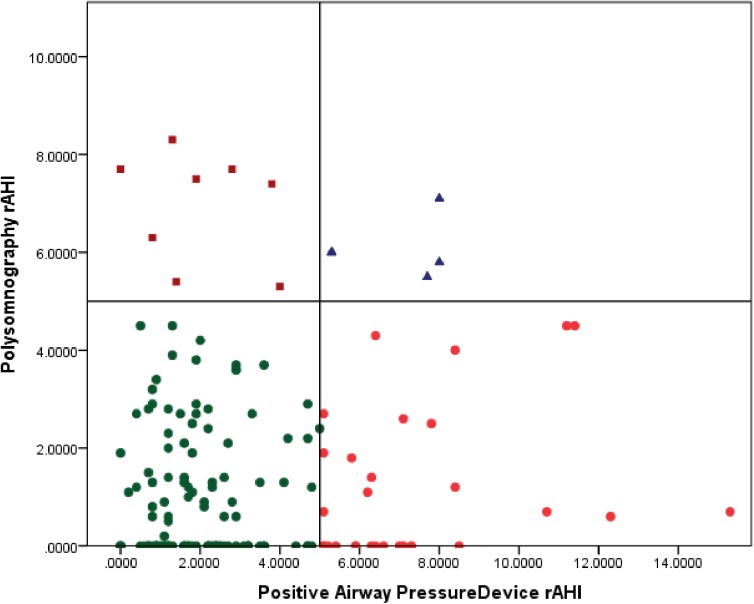
Correlation of apnea–hypopnea indices (AHI) obtained by polysomnography (PSG) and positive airway pressure device (PAP). Green circles represent the category of patients for whom the AHI was ≤5 both in PSG and using the PAP device (n = 121). Red circles represent the category of patients for whom the PSG titration yielded an AHI of ≤5 whereas the PAP device AHI was >5 (n = 29). Blue triangles represent the group of patients for whom both the PSG and PAP titration yielded an AHI of >5 (n = 5). Maroon squares represent the category for whom the PSG titration led to AHI >5 whereas the PAP device AHI was ≤5 (n = 8).

**Fig 2:**
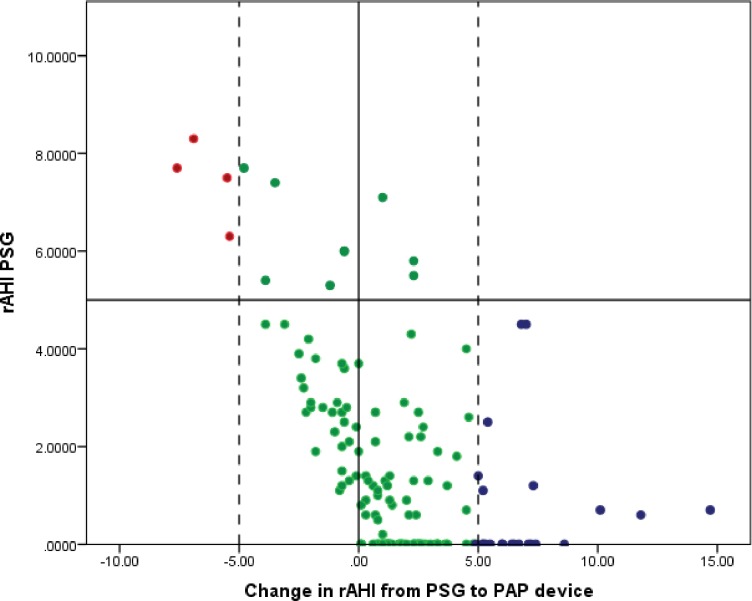
Difference in apnea–hypopnea index (AHI) from polysomnography (PSG) to positive airway pressure device (PAP).

Spearman’s coefficient of rank for the estimated AHI from the PSG and PAP device was not statistically significant (r = 0.128, *P* = 0.102, n = 163). Contextually, an absolute value correlation is less relevant clinically; for example, if PAP titration AHI is 0.1 and the device AHI is 2.1, the absolute value correlation is poor but clinically it still suggests optimally controlled OSAS. To better understand our data in clinical context we classified patients based on clinically relevant subgroups (as AHI ≤5 and AHI >5). This classification is based on the AASM recommendations for optimal CPAP titration [6]. (Fig 1). The majority of patients (80.7%) with PSG AHI of ≤5 had PAP device AHI ≤5 as well. By contrast, if PSG AHI was >5, 61.5% patients reported good control, with AHI ≤5 on PAP device AHI (Table 3). We also looked at the change in AHI from the PSG to the PAP device as shown in Fig 2. Of 163 patients, 138 (84.7%) had a change in AHI of ± 5; and 25 (13.1%) had a change in AHI of > 5 (Fig 2).

**Table 3:** 2 × 2 table for PSG and PAP device AHIs,

			PSG AHI		Total
			0–5	>5	
PAP device AHI	0–5	Count (%)	121 (80.7)	8 (61.5)	129 (79.1)
	>5	Count (%)	29 (19.3)	5 (38.5)	34 (20.9)
Total		Count (%)	150 (100)	13 (100)	163 (100)

Each cell represents the number of subjects. PAP, positive airway pressure; PSG, polysomnography; AHI, apnea–hypopnea index.

For calculating sensitivity and specificity, we considered patients with PSG AHI >5 as the “ill” group and PAP AHI >5 as a “positive” test on the PAP device. The sensitivity (proportion of true positives correctly identified as such) was 0.39 and the specificity (proportion of true negatives correctly identified as such) was 0.81. Thus we found a substantial number of false-positive results (defined as patients whose PSG AHI was ≤5, but the PAP device indicated a value of >5) but a relatively smaller percentage of false-negative results (defined as patients in whom PSG showed a value of >5, but the PAP device data showed AHI of ≤5) (Table 3).

### Subgroup analysis

We performed subgroup analysis of the data specifically looking at the group in which the PSG AHI was ≤5, but the device AHI was >5. We found a statically significant association for the following variables: sex (*P* = 0.017), age (*P* = 0.04), and prescribed PAP pressure (*P* = 0.003). No statistical differences were found for race (*P* = 0.81), BMI (*P* = 0.88), severity of OSA (*P* = 0.164), CPAP vs. BPAP (*P* = 0.562), device manufacturer (*P* = 0.445), or average daily usage of the device (*P* = 0.252).

## Discussion

This study examined two main questions relevant to an AHI reported by a PAP device when used in a constant pressure setting. First, it looked at the real world AHI statistics in patients who have been using the CPAP machine and second, the study evaluated the correlation of the AHI between in-lab CPAP titration and the average AHI reported by the PAP device.

We found that in the majority (81%) of the patients in whom PSG titration was graded as “optimal” based on AASM guidelines (AHI ≤5), the PAP device also reported optimal control with AHI ≤5. From a clinical perspective, this data is meaningful. Typically, the patient returns for the first visit after initiation of PAP therapy. If an optimal titration is achieved in the laboratory, based on our data, the chances of the patients’ smart card AHI of <5 are very high. This suggests continued optimal control of OSA even at home. A clinician may elect to continue the same PAP settings found after the PAP titration.

In a smaller group of patients (19%) of optimally PSG titrated patients, the PAP device reported AHI was suggestive of “good” (AHI >5) control. It will be clinically important to identify the characteristics of this subgroup of patients. While the number of these patients in our study is small, we found no statistical differences among commonly suspected factors such as BMI, severity of OSA, CPAP vs. BPAP, device manufacturer or average daily usage of the device. Three factors however did show statistically significance namely; age, sex and prescribed PAP pressure. Larger studies may identify such subgroup and may have treatment implications.

There are several possible explanations of this finding. Baltzan et al. have studied the prevalence of persistent sleep apnea in patients treated with continuous positive airway pressure[7]. They found that approximately 17% of the patients had persistent sleep apnea despite optimal treatment with single pressure CPAP for at least three months. The second explanation is the differences in the definition of apnea and hypopnea from the PAP device when compared to AASM recommendation. The AASM definition of hypopnea is stable and has shown reproducible scoring and has excellent evidence with regard to the resulting AHI as an independent risk factor for future cardiovascular disease[8]. The PAP definition for hypopnea is variable across systems and is not systematically studied as an independent risk factor for associated cardiovascular mortality. Berry et al. studied the event correlation of AHI determined by a specific manufacturer’s proprietary algorithm with full montage PSG[3]. They found that the agreement of apnea index was quite good, while the agreement of hypopnea index was only fair. It is possible that in our population the PAP device detected higher number of hypopneas which may not have been scored as hypopneas if scored manually based on AASM parameters.

We also report a small group of thirteen patients (8%) in which the PAP titration was graded as “good” according to AASM guidelines (AHI >5 but ≤ 10). We noted that on the follow up visit, the PAP device reported “optimal” control (AHI <5) in eight of these patients. Clinical relevance of this data remains unclear, as the number is small for meaningful statistical interpretation.

Next, we looked at the variance between the two AHI values. We noted that the absolute difference in the PSG and PAP AHI was less than 5 in a majority of cases as well (Fig 2). In a clinical context, for example, if the PSG reported AHI was 0.2, the PAP device AHI was not found to be higher than 5.2 in the majority of cases. Although not a perfect metric, this is again reassuring that for the majority of patients, PAP device reported AHI is not far off from the in-lab AHI.

Finally, to compare the data from our study with the tightly performed previous studies, we calculated the sensitivity and specificity. Our data suggested high specificity 0.81 and lower sensitivity 0.39. These numbers are in similar trend as reported by earlier studies[3,8]. They also reported a higher specificity and lower sensitivity.

Our study has several limitations. It was a retrospective study, with a heterogeneous population and hence is at risk of its inherent biases. Furthermore, we compared AHI from the PAP device after 30 to 90 days of PAP usage with the single night PSG AHI. Can we truly compare the two AHIs in a realistic manner since they are collected temporally apart? To our knowledge, this question is unanswered. Previous studies have shown that CPAP usage after one week can lead to improvement in upper airway anatomy[9]. However, this observation would not account for the smaller percentage of false-negative (PSG AHI >5 with PAP device AHI ≤5) than false-positive (PSG AHI ≤5 with PAP device AHI >5) in our study. We assumed that because we averaged the PAP device smart card data over a prolonged period of usage (average usage 283 ± 142 minutes/day, for an average of 32.4 ± 20 days), the data will be closer to the representative AHI for the individual patient.

In conclusion, we report the real world statistics of PAP device AHI from a university based sleep center and compared it with PSG AHI. Our results suggest that in a majority of patients who were optimally titrated in the sleep laboratory, the PAP device continued to show optimal control at home.

## Supporting information

S1 Dataset Microsoft Excel file containing the dataset of the demographic and apnea hypopnea indices. (XLSX)
